# Attention modulates incidental memory encoding of human movements

**DOI:** 10.1007/s10339-022-01078-1

**Published:** 2022-02-28

**Authors:** Shiau-Chuen Chiou

**Affiliations:** 1 Neurocognition and Action Research Group, Center for Cognitive Interaction Technology (CITEC), Bielefeld University, Inspiration 1, 33619 Bielefeld, Germany; 2 Faculty of Psychology and Sports Science, Bielefeld University, Bielefeld, Germany

**Keywords:** Attention, Memory, Spatiotemporal dependence, Whole-body movement

## Abstract

Attention has been shown to enhance the processing of task-relevant information while suppressing the processing of task-irrelevant information. However, it is less clear whether this attentional modulation exists when there is an intrinsic dependence between task-relevant and task-irrelevant information, such as the dependence of temporal processing on spatial information. In this study, we used complex whole-body movement sequences to investigate the extent to which the task-irrelevant spatial information (trajectory) is processed when only the temporal information (rhythm) is in focus. Moreover, we examined, if the task-irrelevant spatial information is “co-selected” with the target temporal information as predicted by the intrinsic spatiotemporal dependence, whether task-driven attention that is actively directed to spatial information provides extra benefits. Through a two-phase experiment (an incidental encoding phase followed by a surprise memory test phase), we found that the task-irrelevant spatial information was not only perceived but also encoded in memory, providing further evidence in support of a relatively automatic co-selection of spatial information in temporal processing. Nevertheless, we also found that movements whose trajectories were intentionally attended to during the encoding phase were recognized better in the test phase than those that were not, indicating a further modulation from attention on incidental memory encoding and information processing.

**Supplementary Information:**

The online version contains supplementary material available at 10.1007/s10339-022-01078-1.

## Introduction

Attention provides a means of selecting information from the environment that is most relevant to current behavioral goals. Attention can also facilitate sensory processsing (Corbetta et al. 1990; Reynolds and Chelazzi 2004; Yantis and Serences 2003) and enhance memory encoding (Aly and Turk-Browne 2016; Chun and Turk-Browne 2007; Gazzaley and Nobre 2012; Uncapher and Rugg 2009). Although it has been well established that the attended information, either in a particular location or with a specific feature, will be processed more efficiently and better remembered, it is less clear whether the unattended or task-irrelevant information can be excluded from processing, and if yes, to what extent and in which stage (i.e., early perceptual stage or late cognitive stage) (see Driver 2001 for a review).

The load theory of selective attention (Lavie 1995, 2005, 2010; Lavie et al. 2004; see Murphy et al. 2016 for a recent review) suggests that the extent to which task-irrelevant distractors are perceived depends on both perceptual load and cognitive load of the current task. When the perceptual load is high, no spare attentional resources are available for distractors, resulting in performance that is consistent with “early-selection” view of attention (e.g., Broadbent 1958; Treisman 1969). On the contrary, when the perceptual load is low, the spare capacity involuntarily “spills over” to process task-irrelevant information, and thus the suppression of distractors would rely on “late selection”, which is supposed to prevent the perceived task-irrelevant information from entering awareness or gaining control over behavior (e.g., Deutsch and Deutsch 1963; Duncan 1980). Since to actively maintain stimulus-processing priorities requires executive cognitive control, high cognitive load of the current task, such as from working memory, may impair the efficiency of late selection (de Fockert et al. 2001; Lavie 2010).

Although the load theory has been influential since its proposal, there has been evidence showing that perceptual load is not the only factor that determines the selection processes (see Benoni and Tsal 2013; Khetrapal 2010 for critical reviews). Factors such as perceptual grouping or object-based attention may also affect the degree of distractor processing. Specifically, it has been shown that when task-relevant and task-irrelevant information pertain to the same object or perceptual group (through Gestalt cues like continuation or connectedness), the corresponding object-based attention will override the effect of perceptual load and dominate the processing of task-irrelevant information likely through the mechanism of “attentional spreading” (Chen 2003; Cosman and Vecera 2012; Richard et al. 2008). Namely, the task-irrelevant information will be “co-selected” with the target information and subject to attentional modulation irrespective of perceptual load. Note that the inherent properties of a stimulus may further influence how effective irrelevant features of an attended object can be suppressed. For example, Mayer and Vuong (2012) showed that unattended color or motion did not affect perceptual discrimination of attended shape, while unattended shape affected the processing of attended motion. The finding is consistent with the “shape bias” in object recognition (Biederman 1987) and suggests that the shape might be processed automatically (i.e., difficult to suppress) due to its dominant role in object recognition.

A similar asymmetric relationship has been found in the processing of spatial and temporal information (Casasanto and Boroditsky 2008; Casasanto et al. 2010; Dormal and Pesenti 2013; Santiago et al. 2011; Starr and Brannon 2016). For example, Casasanto and Boroditsky (2008) asked participants to reproduce spatial displacement or temporal duration of a visually presented stimulus (i.e., a growing line or a moving dot). Results revealed that participants’ reproduction of duration was consistently interfered by irrelevant spatial information, while their reproduction of displacement remained precise regardless of irrelevant temporal information. The finding indicates that participants were unable to ignore task-irrelevant spatial information when processing the corresponding temporal information, but not vice versa. This line of research suggests that temporal representation may be intrinsically dependent on spatial representation (Casasanto and Boroditsky 2008; Casasanto et al. 2010) or that the spatial information may be processed more automatically than temporal information due to its higher salience in the visual modality (Dormal and Pesenti 2013; Santiago et al. 2011; Starr and Brannon 2016).

Previous research on asymmetric space–time interdependence has provided evidence in support of a relatively automatic co-selection of task-irrelevant spatial information in temporal processing. However, since most research so far has been using simple stimuli (e.g., lines or dots), it is unclear whether this finding can be generalized to more complex stimuli such as whole-body human movements or actions. For one thing, increased complexity of a stimulus may impose additional demand on both perceptual and cognitive systems and thus change the way the stimulus is processed; for another thing, the possibility of forming an abstract higher-order representation from complex stimuli may decrease the reliance of temporal representation on spatial information.

As human movements unfold over time, the spatial and temporal information can be viewed as intrinsically integrated at an early stage of processing. For example, a continuous movement percept can be formed by integrating the “snapshots” of body shapes over time (Giese and Poggio 2003; Lange et al. 2006). At a higher level, temporal information can also be defined as the change of spatial information over time, such as speed (i.e., the distance travelled along a trajectory divided by elapsed time) or rhythm (i.e., the structure of temporal durations conveyed through a sequence of movements). Based on this definition, it is reasonable to hypothesize that the processing of temporal information would rely, to some extent, on spatial information as being second-order features (but not vice versa), and that the task-irrelevant spatial information would still be processed when only the temporal information is in focus. What is less clear, however, is to what extent this co-selection might occur. Does it merely occur in the perceptual level, or it might go beyond that into cognitive processes such as memory? It is also unclear, if the spatial information, albeit task-irrelevant, is likely to be co-selected with the target temporal information, whether *additional* attention that is *actively* directed to the spatial information (due to task relevance) provides extra benefits.

In the current study, we used complex whole-body movement sequences as visual stimuli to investigate the extent to which the task-irrelevant spatial information (movement trajectory) is processed when only the temporal information (movement rhythm) is task-relevant. We also examined how the active attention to spatial information (as opposed to unintended co-selection) might influence its level of processing as measured by the long-term retention in memory. We designed a two-phase experiment including an incidental encoding phase and a surprise memory test phase. During the encoding phase, participants performed a change detection task (same/different judgment) on whole-body movement sequences with two foci of attention: (1) *Temporal-only* and (2) *Both*. In the *Temporal-only* condition, only temporal changes could occur and thus the spatial information was task-irrelevant; while in the *Both* condition, the change could occur in either spatial or temporal domain and thus both types of information were task-relevant. Note that the “change” we referred to in the current study was the change of the whole sequence (i.e., movement trajectory or movement rhythm of the sequence, see “Method”) rather than the change of a single unit of the sequence.

Importantly, to better probe the effect of task-driven attention on spatial processing as opposed to the effect of “co-selection” or “attentional spreading”, in which spatial information is automatically processed with the target temporal information, we used a stimulus set of which the processing demand of spatial information was low (i.e., participants’ sensitivity to spatial changes was high) to minimize the task-driven redistribution of attentional resources between spatial and temporal domains. Since the processing demand of temporal information was relatively high (i.e., participants’ sensitivity to temporal changes was relatively low), it is assumed that any re-allocation of attentional resources from temporal to spatial processing in the *Both* condition would impair temporal performance, as no spare capacity was available. This might further result in qualitative changes of processing strategy between the *Temporal-only* and *Both* conditions, making the effect of attentional focus less comparable.

The encoding phase was followed by a 10-min break and then the surprise memory test phase wherein participants performed a recognition task (old/new judgment) on movement segments extracted from observed or new sequences. Note that the “surprise” means that participants were not informed about the memory test in advance and thus had no incentive to actively memorize the movements observed in either the *Temporal-only* or the *Both* condition. We used the recognition performance as a key indicator to examine whether the task-irrelevant spatial information is encoded in memory and whether the *additional* task-driven attention on spatial information during the encoding phase provides extra benefits to its long-term retention. Given the dependence of temporal processing on spatial information observed in previous research, we predicted that the task-irrelevant spatial information would be co-selected and processed. Nevertheless, active attention might further enhance the processing of the attended information.

## Method

### Participants

Forty participants were recruited for the experiment. Two were excluded from analyses due to below-chance performance (out of 2 standard deviations from the group mean), leaving a final sample of 38 participants (23 female; aged 18–40 years, *M* = 24.8, SD = 4.0). A minimum sample size of 34 was determined based on a power analysis (using G*Power 3.1; Faul et al. 2007) to provide a power of 0.80 at an alpha level of 0.05 to detect medium effects (*d* = 0.5) for within-subjects comparisons of performance under two experimental conditions (*Temporal-only* vs. *Both*). Participants were naïve to the purpose and the design of the experiment. None of them have viewed the experimental materials before.

Participants’ experiences in dance, music, and sport were evaluated by a questionnaire and reported here as expertise indexes (0: No experience, 1: Beginner, 2: Intermediate amateur, 3: Advanced amateur, 4: Professional) of 0.5 (SD = 0.8), 0.7 (SD = 0.8), and 1.4 (SD = 1.1), respectively, defined by both the training length and skill level.^1^ No professionals were recruited. Participants signed informed consent prior to the experiment and received €8 per hour for their participation. The study was conducted in accordance with the ethical principles stated within the declaration of Helsinki (1964) and was approved by the Ethics Committee of Bielefeld University.

### Stimuli and apparatus

Thirty-eight whole-body movement sequences selected from a stimulus pool originally created for another study (unpublished data)^2^ were used in the current experiment, of which 30 sequences were used in the incidental encoding phase and all 38 sequences were used in the surprise memory test phase (see “Procedure” and Table 1).

**Table 1:** Trial distribution and its relation to movement sequences

	Number of sequences	Incidental encoding phase: change detection task			Surprise memory test phase: recognition task		
		Number of trials			Number of trials		
		Same	Different	Total	1-unit	2-unit	Total
Temporal-only	12	24	24	48	24	12	36
Both_temporal_	12	24	24	48	24	12	36
Both_filler_	6	24	24	48	12	6	18
New	8				16	8	24
Total	38				76	38	114

In the *Both* condition of the change detection task, sequences with potential spatial changes, i.e., spatial fillers (Both_filler_), were displayed twice as frequent as those with potential temporal changes (Both_temporal_)

#### Movement design

**Fig. 1:**
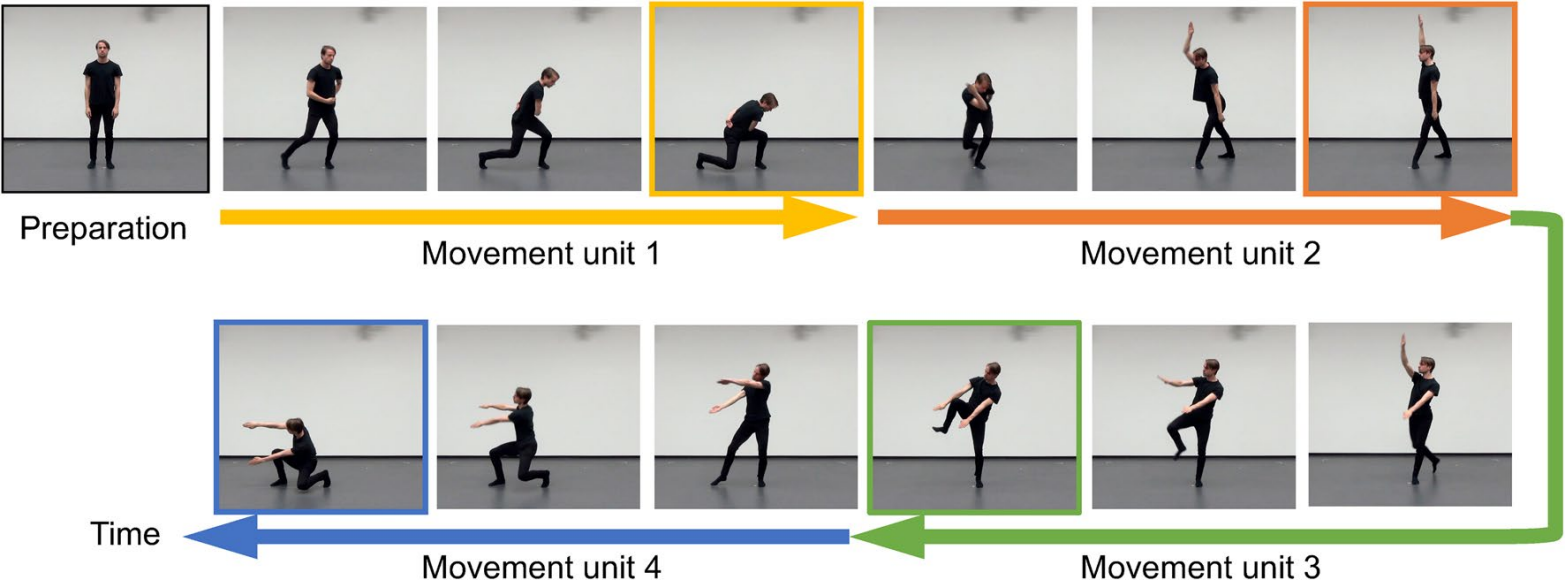
Illustration of a whole-body movement sequence. Movement sequences started at the center of the recording region with a relaxed standing pose, followed by four linked movement units, i.e., the ending pose of the first unit was the starting pose of the second one, and so on. Movements were all without interpretable external goals and action semantics (see Online Resource 1–3 for sample video clips)

Each sequence started with a relaxed standing pose (i.e., feet were shoulder width apart and arms were at the sides of the body), followed by four movement units. A movement unit was defined as a coordinated whole-body movement that can be performed with a bell-shaped velocity profile (i.e., accelerating till the midpoint of the movement and then decelerating) (Abend et al. 1982).^3^ Thus, each movement unit had a clear starting point and ending point where the velocity was zero. Moreover, to better resemble the continuous nature of real-world human movements, the ending pose of the first unit was linked with the starting pose of the second one, and so on, to create a relatively continuous trajectory. In other words, each unit of a sequence had its own velocity profile, movement pattern, and use of body parts, but formed a continuous trajectory when aligned in a specific order (see Fig. 1). Movement sequences were performed by two professional dancers (half by a female dancer and the other half by a male dancer, both in fitted black clothing) at the center of a 3 m × 3 m recording region with a maximum radius of approximate one step outward from the center point. In addition, movements were all without interpretable external goals and action semantics to diminish the influence from long-term semantic memory.

#### Rhythm design

Each movement sequence was performed in four different rhythms (3212, 2132, 1223, and 2321). All were eight beats in a 4/4 musical meter and paced at a tempo of 90 beats per minute, yielding a sequence length of about 6 s after including one additional beat for preparation. Each rhythm was composed of four temporal durations, one 1-beat duration (1), two 2-beat durations (2), and one 3-beat duration (3), corresponding to four movement units of a sequence. When the same trajectory was performed, a shorter duration also implied a higher speed. In addition, the four rhythms used in the current study were all metric complex rhythms (i.e., integer-ratio rhythms without regular temporal accents aligned with the beat), which were shown to be more difficult to induce beat perception than metric simple rhythms, such as 3122 or 1313 (Grahn 2012). Metric complex rhythms were used to avoid beat-based encoding. Note that positions of the shortest (1-beat) and the longest (3-beat) durations were balanced to take potential salience effects into account; total duration difference (by unit) between each of the two rhythms were the same with the smallest duration difference (i.e., one beat or 667 ms) much longer than the temporal resolution of the visual modality (~ 100 ms).

#### Video recording, post-editing, and display

Movement recordings were made with a digital video camera recorder (Sony HDR-CX430V) at 50 frames per second against a white background and a gray floor. The dancers performed each movement sequence in four different rhythms for at least two times with a metronome to ensure the consistency of trajectory and the precision of rhythm across different recordings. Videos were then edited on a frame basis using the software iMovie (Apple, Inc.) and presented silently (i.e., without the sound of metronome) to participants at 1600 × 900 pixels on a 24-inch LCD screen (Dell U2412M) with a viewing distance of ~ 50 cm. The experimental flow and data analysis were programmed in Python, and stimuli presentation was implemented with the PsychoPy software package (Peirce 2007, 2008).

#### Stimulus validation

As mentioned previously, we intended to use a stimulus set of which the processing demand of spatial information was low to better probe the effect of task-driven attention on spatial information as opposed to the side effect (i.e., co-selection) resulting from temporal processing. The characteristic of the stimulus set had been validated by one of our previous studies (*N* = 26, unpublished data) wherein participants performed a similar change detection task as the one used in the current study. Results revealed that performance (measured by proportion correct) of detecting temporal changes of a four-unit sequence was about 70%, while performance of detecting spatial changes was nearly perfect when both types of information were task-relevant.

### Procedure

#### Incidental encoding phase

In the incidental encoding phase, participants performed a change detection task on whole-body movement sequences with two foci of attention: (1) *Temporal-only* and (2) *Both*. In the *Temporal-only* condition, they were instructed to attend to the movement speed to detect temporal changes; in the *Both* condition, they were instructed to pay additional attention to the movement path as there would be spatial changes as well. These two experimental conditions were performed in two separate sessions with an order counterbalanced between participants. The words *path* and *speed* (instead of *trajectory* and *rhythm*) were used in verbal instructions together with simplified animations illustrating these two types of differences (i.e., a square moving along a straight line or a curved line on a computer screen to illustrate a difference in “path”, and a square moving along a straight line with a low or a high speed to illustrate a difference in “speed”) to make the concepts more understandable to participants.

In the *Temporal-only* condition, the sample sequence and the test sequence were either the same (i.e., with the same trajectory and the same rhythm) or different in rhythm (i.e., same in trajectory). For example, the sequence A_3212 (with trajectory A and rhythm 3212) would form a *same* trial with the sequence A_3212 and a *different* trial with the sequence A_2132 (with trajectory A and rhythm 2132). As there were 4 pre-defined rhythms used in the current study, 6 types of temporal contrast (irrespective of order) can be formed for *different* trials. Note that for 2-unit sequences (see below), temporal contrast of 23 vs. 32 was not included due to less salience (i.e., more difficult to discriminate). In the *Both* condition, the sample sequence and the test sequence could be the same, or different in *either* rhythm (i.e., same in trajectory) or trajectory (i.e., same in rhythm), such as A_3212 vs. B_3212 (sequences with different trajectories A and B, but the same rhythm 3212). Three sample video clips (A_2132, A_3212, B_3212) are provided in Online Resource 1–3.

The sequence length was also manipulated (as being 2 or 4 units) to examine whether the memory load might affect memory performance, and if yes, as predicted by the well-documented set-size effect of working memory (i.e., short sequences are memorized better than long sequences) and illustrated by one of our previous studies (unpublished data), how this factor might influence the processing strategy (e.g., the allocation of attentional resources) when observing spatial and/or temporal information of whole-body movement sequences.

**Fig. 2:**
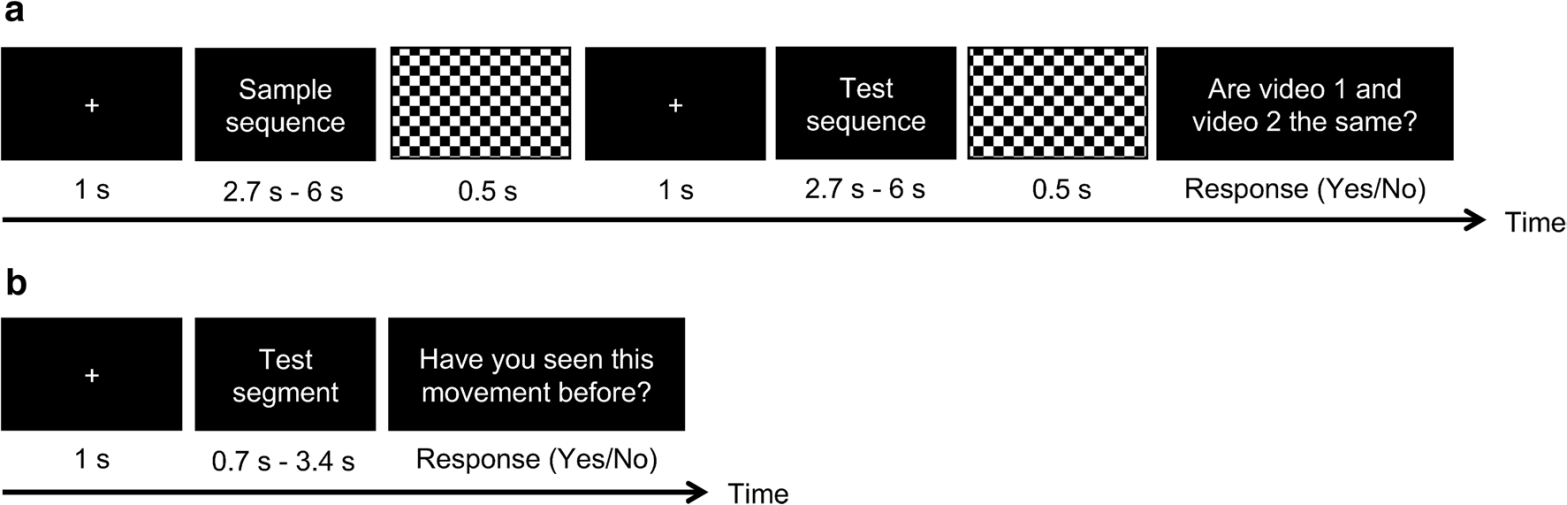
Trial structure of the change detection task (same/different judgment) used in the incidental encoding phase (**a**) and the recognition task (old/new judgment) used in the surprise memory test phase (**b**). In the encoding phase, a sample sequence and a test sequence with the same length of 2 or 4 units were displayed sequentially with a delay of 1.5 s in between (including a mask for 0.5 s and a 1-s fixation cross before the display of the test sequence). Participants were required to detect changes in either temporal domain (“*Temporal-only*” condition) or in both spatial and temporal domains (“*Both*” condition). In the test phase, movement segments of 1 or 2 unit(s) extracted from observed or new sequences were used as test stimuli. Participants were required to make judgments on whether they have seen the movement before

Each trial began with a 1-s fixation cross (+) followed by a sample sequence of 2 or 4 units. To control for the starting pose, a 2-unit sequence was defined as the first-half of a 4-unit sequence (i.e., unit 1 + unit 2). A mask was presented for 0.5 s after the offset of the sample sequence. Following the mask, another 1-s fixation cross was presented before the display of the test sequence (in the same length as the sample sequence, i.e., 2 or 4 units), yielding a retention interval of 1.5 s. After the offset of the test sequence, a mask for 0.5 s was presented, followed by a question “*Are video 1 and video 2 the same?*” Participants then made a yes/no judgment by keystroke on a standard computer keyboard (“F” key and “J” key, respectively, marked in red) (Fig. 2a). Before the start of each session, participants completed 8 practice trials with movement sequences not used in the formal experiment. In practice trials, participants were allowed to replay the sample sequence before making a judgment and received feedback (as shown by the word *correct* or *incorrect* on the computer screen) on a trial-by-trial basis. To ensure participants’ understanding of the task, incorrect trials would be replayed once automatically with an oral check by the experimenter if necessary. No video replay or feedback was provided in formal experiment.

Twenty-four movement sequences pre-selected from the stimulus set were randomly divided into the *Temporal-only* and the *Both* conditions (12 sequences each) on individual-subject basis. Temporal changes would only occur on these sequences. Six additional sequences were used as “fillers” in the *Both* condition for displaying potential changes in the spatial domain. In order to present an equal number of same/different trials as well as trials with spatial/temporal changes, spatial fillers were displayed twice as frequent as sequences with potential temporal changes. Overall, participants completed 48 trials in the *Temporal-only* condition (with 50% different trials) and 96 trials in the *Both* condition (with 50% different trials equally distributed to spatial and temporal changes) (see Table 1). The entire encoding phase lasted about 45 min including short breaks within and between sessions.

#### Surprise memory test phase

After completing two separate sessions (i.e., *Temporal-only* and *Both*) in the encoding phase, participants were asked to take a 10-min break to “refresh themselves”. They were informed that there would be another task after the break, and they were allowed to use the break for their own purposes (e.g., checking the phone calls, going to the restroom, etc.). The experimenter could also have a small talk with participants, while an experiment-related discussion was carefully avoided. The goal of this arrangement was to create a natural segmentation to lower participants’ expectation that the coming task might have a relation with the previous one. After the break, participants performed a surprise movement recognition task, in which movement segments (see below) extracted from 30 sequences observed in the encoding phase as well as 8 new sequences (designed and recorded under the same rules and condition as the old sequences) were used as test stimuli. It would be ideal if a balanced design could have been used, in which the number of *old* trials was equal to the number of *new* trials. However, given the stimulus pool we had (i.e., the one created for a previous study, see Footnote 2) and the difficulty of recording additional movement sequences, especially when low-level visual differences across different recordings were considered, we decided to accommodate our experimental design to the resources we had.

During the test, each movement unit of a sequence was displayed once (and only once) as either a 1-unit segment or as part of a 2-unit segment (i.e., the first half, unit 1 + unit 2, or the second half, unit 3 + unit 4, of the original 4-unit sequence), which was randomly decided for each participant. As movement units were equally divided into these two types of segments, the number of 1-unit trials was twice as many as the number of 2-unit trials (see Table 1). We manipulated the segment length to test if 2-unit segments were recognized better than 1-unit segments. If the movement recognition was mediated by unit-based retrieval, the probability of “encountering” a remembered unit should be higher when observing 2-unit than 1-unit segments, leading to a better performance. Even if the recognition relied on sequence-based representations, we predicted that longer segments, which were supposed to provide additional contextual or ensemble information, would also be easier to recognize.

In addition, test segments varied in movement speed as did original sequences. Rhythms (or temporal durations) that accompanied test segments were the rhythms that accompanied the original 4-unit sequences in the *same* trials of the change detection task. Therefore, the distribution of temporal durations across all segments reflected the original design of the rhythms (see “Rhythm design”); namely, the number of 2-beat units was twice as many as the number of 1-beat or 3-beat units.

Each trial began with a 1-s fixation cross (+), followed by a test segment and then a question “*Have you seen this movement before?*” Participants were asked to make a yes/no response based on the “movement” itself, irrespective of the speed (Fig. 2b). In total, 114 trials were completed (with 90 old segments from 30 old sequences and 24 new segments from 8 new sequences) (see Table 1). No practice trials were provided in this phase. The recognition task took about 8 min to complete.

### Data analyses

Based on the signal detection theory (SDT) (Green and Swets 1966; Macmillan and Creelman 1991), we used the proportion of correct responses, defined as an average of hit rate (correctly responding “same” on *same* trials or “old” on *old* trials) and correct-rejection rate (correctly responding “different” on *different* trials or “new” on *new* trials), as well as the sensitivity measures *d′* and *A′* (Stanislaw and Todorov 1999), where response biases are taken into account, to evaluate participants’ performance in the change detection task and the recognition task.

As we intentionally used a stimulus set of which participants’ sensitivity to spatial changes was much higher than that to temporal changes, this sensitivity difference should be considered when calculating domain-specific performance measures under the *Both* condition (i.e., when two feature dimensions were involved in the decision space). Specifically, an overall hit rate obtained from the *same* trials reflected an evaluation of evidence distributed across spatial and temporal dimensions. Given the high discriminability of spatial information in the current study, the overall hit rate may highly underestimate participants’ performance in the spatial domain. To solve this problem, we used an adapted approach inspired by Luan et al. (2011), combining the SDT analysis with a two-cue fast-and-frugal tree (FFT) to calculate domain-specific hit rates and false alarm rates (incorrectly responding “same” on *different* trials, i.e., complementary to correct-rejection rates) when both spatial and temporal information were task-relevant.

**Fig. 3:**
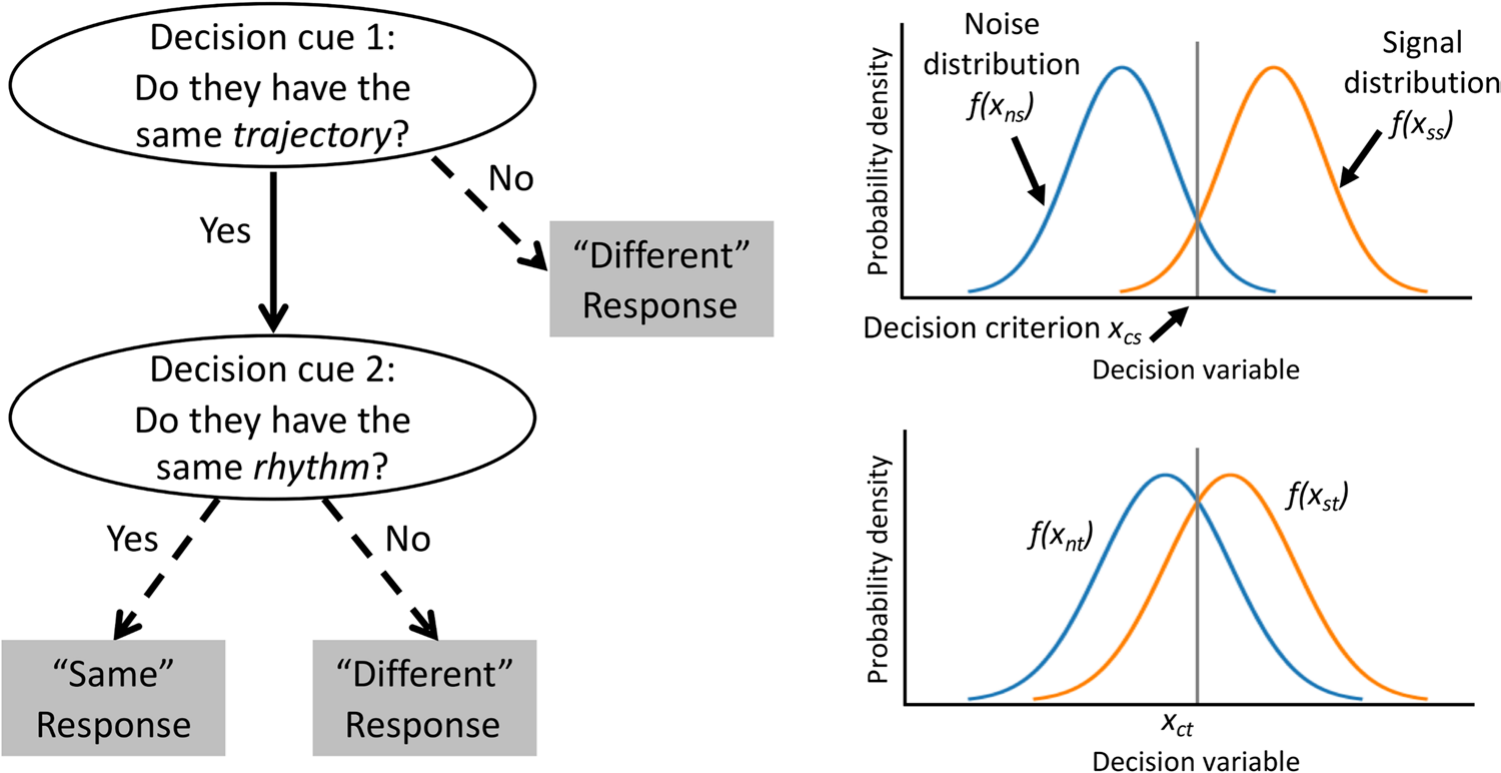
A two-cue fast-and-frugal tree (FFT) and two sets of signal and noise probability density distributions according to the signal detection theory (SDT). The discriminability of spatial information (trajectory) is assumed to be higher than that of temporal information (rhythm), and thus the spatial cue is more likely to be used as the first decision cue. Decision criteria are placed where the noise and signal distributions intersect for simplicity, while each cue may have its own decision criterion, which is not necessarily to be unbiased. *f(x*_*ss*_*)* and *f(x*_*ns*_*)* are signal and noise distributions in the spatial domain; *f(x*_*st*_) and *f(x*_*nt*_*)* are signal and noise distributions in the temporal domain; *x*_*cs*_ and *x*_*ct*_ are decision criteria in the spatial and temporal domains, respectively. Dashed arrows indicate that a final decision is made

In tasks where a binary decision needs to be made with *m* decision cues available, an FFT is defined as a decision tree that has *m* + 1 exits, with one exit for each of the first *m* – 1 cues and two exits for the last cue to ensure that a final decision will be made (Luan et al. 2011). In the *Both* condition of the change detection task, there were two decision cues embedded in the question “*Are video 1 and video 2 the same?*”, namely “*Do they have the same trajectory?*” and “*Do they have the same rhythm?*” A two-cue FFT can thus be constructed based on these two decision cues and a pre-defined 2s (signal)–1n (noise) exit rule, meaning that participants were required to make a “same” judgment when *both* trajectory and rhythm were the same (2s), but a “different” judgment when *either* trajectory or rhythm was different (1n) (see Fig. 3). This decision rule was made explicit and clearly emphasized in verbal instructions. In addition, as the discriminability of spatial information (trajectory) was assumed to be higher than that of temporal information (rhythm), participants were expected to make judgments based on the spatial cue first. But since there were only two cues in this FFT, the cue order would not influence the sensitivity of the FFT.

The overall hit rate calculated from the *same* trials (*P*(Hit)_same_) and the false alarm rates calculated from the *different* trials with spatial (*P*(FA)_spatial_) or temporal (*P*(FA)_temporal_) changes can be expressed as the following:

1 $$P({\text{Hit)}}_{{{\text{same}}}} = P\left[ {\left( {x_{{{\text{ss}}}} > x_{{{\text{cs}}}} } \right) \cap \left( {x_{{{\text{st}}}} > x_{{{\text{ct}}}} } \right)} \right] = {\text{ Hit}}_{{\text{s}}} \times {\text{Hit}}_{{\text{t}}}$$

2 $$P({\text{FA}})_{{{\text{spatial}}}} = P[\left( {x_{{{\text{ns}}}} > x_{{{\text{cs}}}} } \right) \cap \left( {x_{{{\text{st}}}} > x_{{{\text{ct}}}} } \right)] = {\text{FA}}_{{\text{s}}} \times {\text{Hit}}_{{\text{t}}}$$

3 $$P({\text{FA}})_{{{\text{temporal}}}} = P[\left( {x_{{{\text{ss}}}} > x_{{{\text{cs}}}} } \right) \cap \left( {x_{{{\text{nt}}}} > x_{{{\text{ct}}}} } \right)] = {\text{ Hit}}_{{\text{s}}} \times {\text{FA}}_{{\text{t}}}$$

where *x*_ss_ and *x*_ns_ are decision variables in the spatial domain when drawn from signal or noise category; *x*_st_ and *x*_nt_ are decision variables in the temporal domain when drawn from signal or noise category; *x*_cs_ and *x*_ct_ are decision criteria in the spatial and temporal domains, respectively. The hit rates and false alarm rates of the two decision cues are denoted as Hit_s_, FA_s_ (spatial domain), and Hit_t_, FA_t_ (temporal domain). In addition, since incorrect judgments in both *same* and *different* trials mainly resulted from the same inaccurate working memory representation of the spatial and/or temporal information of the sample sequence, we made an additional assumption that participants’ miss rates (incorrectly responding “different” on *same* trials, i.e., complementary to hit rates), denoted as “Miss”, were proportional to their false alarm rates in the respective domains:

4 $${\text{Miss}}_{{\text{s}}} :{\text{Miss}}_{{\text{t}}} = (1 - {\text{Hit}}_{{\text{s}}} ):(1 - {\text{Hit}}_{{\text{t}}} ) = {\text{FA}}_{{\text{s}}} :{\text{FA}}_{{\text{t}}}$$

If FA_s_ = FA_t_ = 0, Hit_s_ and Hit_t_ would be unsolvable. Under this condition, if *P*(Hit)_same_ = 1, it was assumed that Hit_s_ = Hit_t_ = 1 (i.e., perfect discrimination); if *P*(Hit)_same_ < 1, false alarm rates of a similar within-subjects category would be used as an approximation. For example, the ratio of FA_s_ to FA_t_ of 4-unit sequences would be used as an approximation for that of 2-unit sequences. If no within-subjects data were available, a group average would be used instead. By solving Eqs. (1)–(4), hit rates and false alarm rates for spatial and temporal information (i.e., Hit_s_, FA_s_, and Hit_t_, FA_t_) as well as the proportion correct and the sensitivity measures *d′* and *A′* of respective feature dimensions can be calculated.

We set the statistical threshold of Type I error at α = .05 and reported Cohen’s *d* and partial eta squared (η_p_^2^) to indicate effect size. *Post hoc* analyses were conducted using Bonferroni correction.

## Results

### Incidental encoding phase

We first checked if participants’ discrimination performance for spatial changes was high as expected. The results revealed a proportion correct of 98.1% (SD = 3.94%) for 2-unit sequences and 96.1% (SD = 7.06%) for 4-unit sequences when both spatial and temporal information were task-relevant. The close-to-ceiling performance indicates that the processing demand of spatial information was low and thus the *additional* processing demand of spatial information might not decrease the amount of attentional resources that were available for temporal processing. To verify this prediction and to check if the order of performing the two experimental sessions (i.e., starting with the *Temporal-only* condition, followed by the *Both* condition, or the other way around) might also affect performance, we conducted a 2 (focus of attention: temporal-only, both) × 2 (sequence length: 2-unit, 4-unit) × 2 (order: temporal-only + both, both + temporal-only) mixed analysis of variance (ANOVA) on temporal performance (measured by proportion correct), with “focus of attention” and “sequence length” as the within-subjects factors and “order” as the between-subjects factor.

**Fig. 4:**
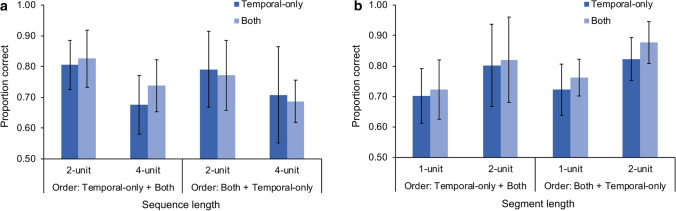
Temporal performance of the change detection task in the incidental encoding phase **(a)** and recognition performance in the memory test phase (spatial fillers were excluded) **(b)** Error bars indicate one standard deviation of the mean

Descriptive statistics of each performance measure under respective experimental conditions are summarized in Table 2. The ANOVA yielded a significant two-way interaction between focus of attention and order, *F*(1, 36) = 4.84, *p* = 0.034, η_p_^2^ = 0.12. Pairwise comparisons (collapsing sequence length) indicated that participants’ sensitivity to temporal changes remained the same irrespective of whether they attended to the temporal information only (*M* = 75.0%, SD = 12.3%) or additionally also to the spatial information (*M* = 73.0%, SD = 8.20%) if they started with the *Both* condition, *t*(19) =  −0.88, *p* = 0.390, *d* =  − 0.20. Surprisingly, for participants who started with the *Temporal-only* condition, their performance improved in the second session when additional spatial information was required to be processed (*M* = 78.1%, SD = 6.74% in the *Both* condition; *M* = 74.1%, SD = 7.36% in the *Temporal-only* condition), *t*(17) = 2.53, *p* = 0.021, *d* = 0.60, illustrating a potential learning effect (Fig. 4a).

**Table 2:** Performance of the change detection task in the incidental encoding phase by order, sequence length, and focus of attention

	Order: temporal-only + both		Order: both + temporal-only	
	2-unit	4-unit	2-unit	4-unit
*Temporal-only* condition				
*P*(Hit)_same_	.83 (.12)	.64 (.19)	.82 (.16)	.70 (.21)
*P*(FA)_temporal_	.22 (.13)	.29 (.15)	.23 (.18)	.28 (.18)
Proportion correct	.81 (.08)	.68 (.10)	.79 (.12)	.71 (.16)
*d′*	1.87 (0.62)	1.03 (0.59)	1.84 (0.85)	1.27 (1.01)
*A′*	.88 (.06)	.75 (.12)	.86 (.11)	.78 (.18)
*Both* condition				
*P*(Hit)_same_	.83 (.14)	.73 (.13)	.82 (.14)	.69 (.16)
Hit_s_	.98 (.04)	.95 (.08)	.99 (.02)	.96 (.08)
Hit_t_	.84 (.14)	.77 (.12)	.83 (.14)	.71 (.15)
*P*(FA)_spatial_	.03 (.06)	.03 (.05)	.02 (.03)	.03 (.05)
FA_s_	.03 (.07)	.04 (.06)	.02 (.04)	.03 (.07)
*P*(FA)_temporal_	.19 (.13)	.29 (.17)	.28 (.21)	.33 (.20)
FA_t_	.19 (.13)	.30 (.16)	.29 (.21)	.34 (.20)
Proportion correct				
Spatial	.97 (.05)	.96 (.07)	.99 (.02)	.96 (.07)
Temporal	.83 (.09)	.74 (.08)	.77 (.11)	.69 (.07)
*d′*				
Spatial	3.49 (0.58)	3.29 (0.75)	3.67 (0.25)	3.40 (0.74)
Temporal	2.10 (0.81)	1.38 (0.52)	1.75 (0.81)	1.13 (0.46)
*A′*				
Spatial	.99 (.03)	.97 (.04)	.99 (.01)	.98 (.04)
Temporal	.89 (.07)	.82 (.07)	.85 (.11)	.78 (.07)

Numbers in the parentheses are standard deviations. *P*(Hit)_same_ is the hit rate calculated from the *same* trials; *P*(FA)_spatial_ and *P*(FA)_temporal_ are false alarm rates calculated from the *different* trials with spatial and temporal changes, respectively. Hit_s_ and Hit_t_ are domain-specific hit rates for spatial and temporal information; FA_s_ and FA_t_ are domain-specific false alarm rates for spatial and temporal information. Proportion correct, *d′*, and *A′* for spatial and temporal information in the *Both* condition were calculated based on the domain-specific hit rates (Hit_s_, Hit_t_) and false alarm rates (FA_s_, FA_t_)

One may suspect that the performance improvement might alternatively indicate that actively attending to spatial information was beneficial for detecting temporal changes. However, if that was the case, participants who started with the *Both* condition should also have performed better in this specific condition, namely that we should have observed a performance decline in the *Temporal-only* condition in this group, but we did not. Moreover, when leaving out potential order effects by comparing the first-session performance of the two order groups, we did not find any significant difference between the *Both* and the *Temporal-only* conditions, *t*(36) =  − 1.05, *p* = 0.299, *d* =  − 0.34 for short sequences; *t*(30.6) = 0.43, *p* = 0.671, *d* = 0.14 for long sequences, indicating that the active attention to spatial information did not benefit temporal discrimination.

Across the two order conditions, as the temporal performance was far from ceiling (e.g., *M* = 79.8%, SD = 10.4% for 2-unit sequences; *M* = 69.3%, SD = 13.0% for 4-unit sequences in the *Temporal-only* condition), it is reasonable to assume that any re-allocation of attentional resources from temporal to spatial processing would have impaired temporal performance, as no spare capacity was available. The results therefore demonstrated that by adding a low demand on spatial processing, we successfully manipulated participants’ focus of attention (through task requirements) while keeping the redistribution of attentional resources at a minimal level. The analysis conducted on the sensitivity measures *d′* and *A′* also led to the same conclusion. The analysis on *d′* again yielded an interaction effect between focus of attention and order, *F*(1, 36) = 4.78, *p* = 0.035, η_p_^2^ = 0.12. Participants who started with the *Temporal-only* condition performed better in the *Both* condition, *t*(17) = 2.69, *p* = 0.015, *d* = 0.63, while no such difference was shown for participants who started with the *Both* condition, *t*(19) =  − 0.75, *p* = 0.461, *d* =  − 0.17. The analysis conducted on *A′* did not reveal a significant effect of focus of attention for both order groups, *F*(1, 36) = 1.92, *p* = 0.175, η_p_^2^ = 0.05.

The main effect for sequence length was significant, proportion correct: *F*(1, 36) = 49.5, *p* < 0.001, η_p_^2^ = 0.58; *d′*: *F*(1, 36) = 51.8, *p* < 0.001, η_p_^2^ = 0.59; *A′*: *F*(1, 36) = 31.3, *p* < 0.001, η_p_^2^ = 0.47; 2-unit sequences were discriminated better than 4-unit sequences, illustrating the set-size effect of working memory. No interaction effect was found between focus of attention and sequence length, proportion correct: *F*(1, 36) = 0.73, *p* = 0.398, η_p_^2^ = 0.02; *d′*: *F*(1, 36) = 0.04, *p* = 0.839, η_p_^2^ = 0.001; *A′*: *F*(1, 36) = 1.89, *p* = 0.178, η_p_^2^ = 0.05, indicating that an increase of cognitive load did not affect the processing strategy or the way attention deployed during observation. No other effects were significant.

### Surprise memory test phase

Recognition performance (measured by proportion correct after considering the false alarm rate obtained from the *new* trials) for movements previously displayed in the *Temporal-only* condition was 71.3% (SD = 8.60%) for 1-unit segments and 81.3% (SD = 10.5%) for 2-unit segments; both were higher than the chance level of 50%, indicating that the task-irrelevant spatial information was not only perceived but also encoded in memory. In addition, to examine whether the task-driven attention provided extra benefits in terms of the long-term retention of the attended information, we conducted a 2 (focus of attention: temporal-only, both) × 2 (segment length: 1-unit, 2-unit) × 2 (order: temporal-only + both, both + temporal-only) mixed ANOVA on recognition performance, with “focus of attention” and “segment length” as the within-subjects factors and “order” as the between-subjects factor. Spatial fillers were excluded from analyses (but see Table 3 for a summary of descriptive statistics). We added “order” as an additional factor to test whether participants might process the observed information in different ways depending on which session they performed at first. Specifically, participants who attended to temporal changes in the first session might bias their attention towards temporal information in the second session (i.e., *Both* condition), resulting in a worse recognition performance in the test phase. On the contrary, participants who attended to both spatial and temporal changes in the first session might process spatial information to a deeper extent even when it was task-irrelevant in the second session (i.e., *Temporal-only* condition), leading to a better recognition performance.

**Table 3:** Performance of the recognition task in the surprise memory test phase by order, segment length, and probe condition

	Order: temporal-only + both		Order: both + temporal-only	
	1-unit	2-unit	1-unit	2-unit
*Hits*				
Temporal-only	.66 (.16)	.75 (.17)	.65 (.17)	.76 (.15)
Both_temporal_	.71 (.15)	.79 (.14)	.73 (.12)	.87 (.12)
Both_filler_	.75 (.22)	.94 (.16)	.84 (.16)	.92 (.10)
*Correct rejection*				
New	.74 (.12)	.85 (.18)	.79 (.14)	.89 (.10)
*Proportion correct*				
Temporal-only	.70 (.09)	.80 (.14)	.72 (.08)	.82 (.07)
Both_temporal_	.72 (.10)	.82 (.14)	.76 (.06)	.88 (.07)
Both_filler_	.74 (.12)	.89 (.16)	.82 (.09)	.91 (.07)
*d′*				
Temporal-only	1.16 (0.55)	1.83 (0.89)	1.33 (0.54)	1.94 (0.49)
Both_temporal_	1.28 (0.62)	1.93 (0.91)	1.56 (0.42)	2.33 (0.52)
Both_filler_	1.49 (0.81)	2.27 (0.92)	1.98 (0.66)	2.36 (0.40)
*A′*				
Temporal-only	.78 (.10)	.86 (.13)	.81 (.08)	.90 (.05)
Both_temporal_	.80 (.10)	.87 (.14)	.85 (.05)	.93 (.05)
Both_filler_	.82 (.11)	.92 (.16)	.89 (.07)	.95 (.04)

Numbers in the parentheses are standard deviations. Performance for Both_filler_ (sequences with potential spatial changes in the *Both* condition, i.e., spatial fillers) is not directly comparable with performance for Both_temporal_ (sequences with potential temporal changes in the *Both* condition), as Both_filler_ were displayed twice as frequent as Both_temporal_ in the incidental encoding phase

The results showed a significant main effect for focus of attention, *F*(1, 36) = 8.75, *p* = 0.005, η_p_^2^ = 0.20; movements whose trajectories were intentionally attended to in the *Both* condition (*M* = 79.6%, 95% CI [76.9%, 82.2%]) were recognized better than those in the *Temporal-only* condition (*M* = 76.2%, 95% CI [73.5%, 79.0%]). The finding indicates a modulation from attention on incidental memory encoding (Fig. 4b). The main effect for segment length was also significant, *F*(1, 36) = 53.6, *p* < 0.001, η_p_^2^ = 0.60; 2-unit segments (*M* = 83.1%, 95% CI [79.9%, 86.3%]) were recognized better than 1-unit segments (*M* = 72.7%, 95% CI [70.3%, 75.2%]), consistent with our prediction that long sequences were recognized better than short sequences. The same conclusion can also be drawn when the analysis was conducted on *d′* and *A′*, respectively. Movement segments previously displayed in the *Both* condition were recognized better than those displayed in the *Temporal-only* condition, *d′*: *F*(1, 36) = 8.25, *p* = 0.007, η_p_^2^ = 0.19; *A′*: *F*(1, 36) = 7.06, *p* = 0.012, η_p_^2^ = 0.16, and 2-unit segments were recognized better than 1-unit segments, *d′*: *F*(1, 36) = 50.0, *p* < 0.001, η_p_^2^ = 0.58; *A′*: *F*(1, 36) = 35.5, *p* < 0.001, η_p_^2^ = 0.50.

The main effect for order was not significant, proportion correct: *F*(1, 36) = 2.01, *p* = 0.165, η_p_^2^ = 0.05; *d′*: *F*(1, 36) = 2.40, *p* = 0.130, η_p_^2^ = 0.06; *A′ F*(1, 36) = 2.93, *p* = 0.096, η_p_^2^ = 0.08, indicating that the performance order did not influence the extent to which the spatial information was processed. Nevertheless, descriptive statistics showed a tendency that participants who started with the *Temporal-only* condition did not recognize as many movement segments from the *Both* condition (measured by hit rate) as those who started with the *Both* condition (see Table 3). The finding suggests that participants who attended to temporal changes first might have biased their attention more towards temporal information in the *Both* condition than those who started with the *Both* condition, despite the fact that no significant difference was shown in the spatial discrimination performance between these two order groups, *t*(36) =  − 0.64, *p* = 0.526, *d* =  − 0.21. However, participants who attended to the spatial information in the first session did not show a similar spatial bias in the *Temporal-only* condition. Their recognition performance for movements from the *Temporal-only* condition was comparable with that of those who started with the *Temporal-only* condition (see Table 3). This difference in processing strategy might result from the difference in sensitivity between spatial and temporal processing. No other effects were significant.

## Discussion

In this study, we used complex whole-body movement sequences to investigate the extent to which the task-irrelevant spatial information (movement trajectory) is processed when only the temporal information (movement rhythm) is in focus. We found that the task-irrelevant spatial information was not only perceived, as predicted by the dependence of temporal processing on spatial information (Casasanto and Boroditsky 2008; Casasanto et al. 2010; Dormal and Pesenti 2013; Santiago et al. 2011; Starr and Brannon 2016), but also encoded in memory, consistent with previous studies showing that durable memory representations can be formed for unattended or task-irrelevant information (e.g., Hutmacher and Kuhbandner 2020; Kuhbandner et al. 2017). In addition, we examined whether attention that is *actively* directed to spatial information provides extra benefits when compared with unintended co-selection or attentional spreading resulting from temporal processing. The answer is yes. We found that movements whose trajectories were intentionally attended to during observation were recognized better in a later memory test than those that were not, indicating a modulation from attention on information processing and incidental memory encoding.

Note that the temporal performance in the incidental encoding phase was not impaired by additional demand from spatial processing, suggesting that a similar amount of cognitive resources were allocated to the temporal processing in the *Both* condition as in the *Temporal-only* condition and that no significant redistribution of attentional resources had occurred between these two experimental conditions. Although there might be a potential difference in processing strategy depending on which session, *Temporal-only* or *Both*, was performed at first, the results indicate that all participants, irrespective of their performance order, tended to allocate a maximum possible amount of attentional resources to temporal processing across the *Temporal-only* and *Both* conditions. For participants who started with the *Temporal-only* condition, it was a bias towards temporal information in the *Both* condition (i.e., restricting the additional spatial processing required by the task to a minimum level); for participants who started with the *Both* condition, it was a selective attention to temporal processing in the *Temporal-only* condition (i.e., without a notable spatial bias). As indicated previously, this difference in processing strategy might result from the sensitivity difference between spatial and temporal processing. It is worth noting that the interaction effect we found between focus of attention and order in the change detection task was not reflected in the memory behavior observed in the recognition task. The finding may suggest that working memory representations and visual cues participants used for detecting changes between two sequentially displayed movement sequences might differ qualitatively from long-term memory representations and retrieval cues they used for recognizing movements.

Moreover, the finding that the additional demand from spatial processing did not impair temporal discrimination also implies that processing temporal information required or “co-occurred” with a certain level of spatial processing, and *this* level of processing was already sufficient to fulfill the task requirement in the spatial domain. Although the finding provides further evidence in support of the dependence of temporal processing on spatial information, it is so far unclear whether temporal information is *fully* integrated with the corresponding spatial information or it is *partially* dependent on it. Previous research proposes that different features of a movement (e.g., action type, duration) are bound together and stored as an integrated representation in working memory (Wood 2007, 2011), consistent with the view of “object-based storage” assuming an automatic integration of features into objects (Luck and Vogel 1997). Nevertheless, the current results showing that the spatial processing can be modulated independently from temporal processing by focus of attention imply that spatial and temporal information, albeit interdependent, might not be fully (and compulsorily) integrated.

If the change of attentional focus did not lead to resource redistribution between spatial and temporal processing, how did attention modulate incidental memory encoding of spatial information? One possibility is, although the same amount of cognitive resources might be allocated to temporal processing in both experimental conditions, different aspects of spatial information might be processed when different attentional foci were adopted. As suggested by Cavanagh (1991), passive motion processing is responsible for low-level motion detection, which is accomplished relatively automatically via parallel arrays of motion detectors, while active motion processing involves the use of attention to track selective features across time. The passive/active distinction therefore implies that information that is extracted by passive motion processing might be fundamentally different from that extracted by active motion processing (Thornton et al. 2002). In the current study, for example, a low-resolution movement configuration might be processed when only temporal judgements were to be made, while active attention might be directed to the movement of specific body parts when additional spatial judgements were to be made. Although these two types of information may be equally beneficial for temporal processing, the latter may provide more cues for movement recognition in the test phase. In other words, spatial features that participants captured and used for the change detection (and the later recognition) task might depend on task requirements or, more specifically, focus of attention.

This explanation resonates with the distinction between attention as mental effort (or resource) and attention as selective processing (Chun and Turk-Browne 2007; Johnston and Dark 1986). Previous studies have shown that implicit learning, for example, can occur independently of attentional load (such as when a cognitive-demanding secondary task is introduced), but requires task-relevant stimuli to be selectively attended (Jiang and Chun 2001; Jiménez and Mendez 1999; Turk-Browne et al. 2005). Our finding also suggests that attention can enhance memory not necessarily through the deployment of additional resources, but through a selective process, in which features that are relevant to task performance are selected for processing. Further studies would be required to examine which types of information are most likely to be extracted under which types of attentional foci and task requirements.

Alternatively, it might be possible that the better recognition performance for movements previously displayed in the *Both* condition was due to a deeper level of information processing required by the task rather than a direct modulation from attention. Specifically, when performing a change detection task, participants need to store the observed information (sample sequence) temporarily in memory before it can be compared with the subsequent information (test sequence). That is, when spatial judgments were required, spatial information was actively stored in memory for a short period of time (retention interval) before the judgments were made. This additional processing may enhance the incidental memory encoding and thus increase the probability of successful retrieval. Although this explanation is plausible, we do not expect large effects from working memory. In one of our previous studies (unpublished data), participants’ performance in a similar change detection task was close to ceiling even with a retention interval of 7 s. Therefore, the working memory demand imposed by a retention interval of 1.5 s in the current study should be low and even neglectable. Moreover, as spatial fillers (movement sequences used in the trials with spatial changes) were excluded from analyses, the better recognition performance for movements displayed in the *Both* condition cannot be attributed to change-induced effects (such as attentional capture) in the spatial domain during the encoding phase.

Although our finding indicates that the task-irrelevant spatial information was not only perceived, but also encoded in memory as illustrated by the above chance recognition performance in the surprise memory test phase, one should note that the current finding is based on an experimental design of which the processing demand of spatial information was low (i.e., spatial discriminability was high) and the processing demand of temporal information was high (i.e., temporal discriminability was low). In other words, the task-irrelevant spatial information might have been processed to a lesser extent if the discriminability of temporal information has been increased or the salience (but not discriminability, see below) of spatial information has been decreased. According to the attentional spreading account of object-based attention, the processing of task-relevant and task-irrelevant information would be enhanced simultaneously by attentional focus if relevant and irrelevant information pertain to the same object (Chen 2003; Cosman and Vecera 2012; Richard et al. 2008). This would predict that an increase of temporal discriminability might decrease the corresponding processing of spatial information due to an overall decrease in attentional demand. Moreover, reducing the salience of task-irrelevant spatial information might also suppress its processing and lessen its chance of being retained in memory.

Note that the “salience” here refers to the characteristics of movements per se rather than the spatial discriminability between two movement sequences. Based on the current design, a decrease of spatial discriminability would be irrelevant to the *Temporal-only* condition and the co-processing of spatial information, as there would be only one same movement sequence (i.e., trajectory) displayed in each trial, either with or without a change in rhythm. A decrease of spatial discriminability, however, would increase the processing demand of spatial information in the *Both* condition, requiring more cognitive resources to be allocated to the spatial domain (i.e., less to the temporal domain when compared with the *Temporal-only* condition). This resource re-allocation is what we tried to avoid (or minimize), as it would make the comparison between the effect of “passive co-selection” and the effect of “active task-driven attention” less interesting, since one can easily attribute the extra benefits to the larger amount of cognitive resources allocated to the attended information in the latter condition.

Furthermore, one should also note that human body and movement is a type of information that is not only complex, but also highly familiar and socially relevant to all human beings. The fact that most people are extremely efficient in biological movement perception and sensitive to body related changes (Blake and Shiffrar 2007; Peelen and Downing 2007) would imply that the task-irrelevant spatial information of human movements might be more difficult to suppress in general than that of other types of complex or dynamic stimuli.

In conclusion, this study provides evidence in support of a relatively automatic co-selection of task-irrelevant spatial information in temporal processing by using more ecologically-valid and complex movement stimuli. Although this finding might be well expected given the intrinsic spatiotemporal dependence in human movements, what we found more interesting is that the unintended co-selection of task-irrelevant information did not prevent active attention from further enhancing its processing. In other words, the task-driven active attention provided additional benefits, expressed as a deeper level of information processing in the current study, over and above the effects from attentional spreading. This independent modulation from attention also suggests that spatial and temporal information of movements, albeit interdependent, might not be fully integrated.

## Supplementary Information

Below is the link to the electronic supplementary material.

Supplementary file1 (MP4 6933 KB)

Supplementary file2 (MP4 6956 KB)

Supplementary file3 (MP4 6747 KB)

## Footnotes

1 The expertise index (0–4) is defined as the following: 0: No experience, 1: Beginner (skill level = 1 or 2 in a five-point scale, or skill level > 2 but training length < 3–5 years), 2: Intermediate amateur (skill level = 3 and training length ≥ 3–5 years, or skill level > 3 but training length < 6 years), 3: Advanced amateur (skill level = 4 and training length ≥ 6 years), 4: Professionals (skill level = 5 and training length ≥ 6 years).

2 A total of 44 whole-body movement sequences were originally designed and recorded for another study conducted by the author, which is currently in preparation and not yet published.

3 There were a few exceptions, such as a jump or a circular movement, that were not performed with a bell-shaped velocity profile. Those movements were included into the stimulus set only if a clear starting point and ending point can be identified and the manipulation of movement speed can be precisely implemented.
